# Persistence of Cellulolytic Bacteria *Fibrobacter* and *Treponema* After Short-Term Corn Stover-Based Dietary Intervention Reveals the Potential to Improve Rumen Fibrolytic Function

**DOI:** 10.3389/fmicb.2018.01363

**Published:** 2018-06-26

**Authors:** Xiao Xie, Chunlei Yang, Le L. Guan, Jiakun Wang, Mingyuan Xue, Jian X. Liu

**Affiliations:** ^1^Institute of Dairy Science, College of Animal Sciences, Zhejiang University, Hangzhou, China; ^2^Department of Agricultural, Food and Nutritional Science, University of Alberta, Edmonton, AB, Canada

**Keywords:** rumen microbiota, dynamics variation, low-quality forage, adaptation, persistence

## Abstract

Limited lignocellulose degradation is the primary obstacle to feed digestion efficiency in ruminant animals. Low-quality forage with high levels of fibrous components can favor the proliferation of fibrolytic bacteria, but whether this can result a profound microbial shift after dietary intervention remains unclear. In this study, we monitored the microbial communities in the rumens of five ruminally cannulated Hu sheep through dietary transition from alfalfa hay (AH, pre-CS) to corn stover (CS, post-CS) and then back to AH (post-AH), with each treatment lasting for 14 days. The CS intervention significantly increased the relative abundance of microorganisms involved in lignocellulose degradation, including *Fibrobacter* and *Treponema*. When the diet was switched back to AH, the microbial community did not completely return to a pre-CS treatment state. In the post-AH microbial community, the relative abundances of *Fibrobacter* and *Treponema* were persistently high, and were similar to those in the post-CS community. Meanwhile, the diversity of the microbial community increased after dietary transition from AH to CS and remained significantly higher after transition from CS to AH compared to those under the original AH diet. Enzyme activity measurement verified significant increase of carboxymethyl cellulase (CMCase) and xylanase catalytic activities in the rumen. Microbial functional predictions using Tax4Fun revealed that this microbial persistence may enhance the carbohydrate metabolism pathway in the rumen. In summary, persistence of *Fibrobacter* and *Treponema* can be enhanced through a low-quality forage intervention at least for 2 weeks, which may enlighten the reprogram of microbial population in the rumen in the future.

## Introduction

The rumen is a complex ecosystem that contains a wide range of functional microorganisms that can degrade feed particles (Huws et al., 2016). The rumen microbes ferment human-inedible plant-based biomass to form volatile fatty acids, providing a major nutrient source for host animal growth. Although the rumen is one of the most effective biosystems to utilize complex fibrous substrates, fiber digestion in the rumen is not optimal due to nature of the fibrous component in many types of forage (Krause et al., 2003). Improving the ability of rumen microbiota to degrade recalcitrant lignocellulose is essential to improving animal performance and is generally highly desirable.

Despite continuous research efforts, strategies aimed at introducing high fibrolytic microorganisms to the rumen have mostly yielded disappointing results (Krause et al., 2001; Chiquette et al., 2007; Ribeiro et al., 2017). The possibility of succession in allochthonous microbes is limited, as results are often inconsistent or short-lived. This limitation is mainly due to the resilience and individuality of the host, especially for the well-established and fully matured rumens of adult animals (Ribeiro et al., 2017). Feed has been considered the main driver in altering the structure and activity of rumen microbiota, which overwhelms the differences of the host’s genetic background in determining microbial community composition (Carmody et al., 2015; Henderson et al., 2016). Evidence in monogastric animals also reveals that the gastrointestinal microbiome patterns formed under long-term dietary regime were persistent (Wu et al., 2011). Specifically, long-term or lifetime consumption of natural high-lignocellulose feedstuffs facilitates higher fiber digestion capacity by wild ruminants such as bison, buffalo, and yak compared to domestic bovines (Mcdowell, 1988; Dai et al., 2012). As such, feed intervention can be a better choice for rumen microbial modification and can sidestep differences caused by individuality and host resilience.

Cereal straw is a rich biomass resource and represents an important forage source for ruminant animals in developing countries. Due to its low nutrient levels, cereal straw is not widely accepted as the main forage for animal production in large-scale farms. Nevertheless, feeding animals with low-quality forage usually stimulates the growth of lignocellulose-utilizing bacteria and improves fibrolytic potential in the rumen (Liu et al., 2016). Since forage usually makes up half or more of the diet of ruminant animals, feeding cereal straw as a regulator to enhance the activity of fibrolytic bacteria can be a strong and sustainable practice.

According to a recently published paper on monogastric animals, despite microorganisms’ ability to rapidly adapt to the new diet, some taxa can nonetheless memorize previous dietary exposure and persist under the new diet as a result (Thaiss et al., 2016). Although many attempts have been made to identify the effects of forage type on the microbiome in the rumen, most of them are still superficial with regards to comparing microbial communities sampled at specific points in time (Liu et al., 2016; Wang et al., 2016). The microbiome reaction to the post-dietary treatment is usually neglected: therefore, how the previous dietary intervention affects the overall rumen microbiome is unclear.

In this study, we used ruminally cannulated sheep as models to investigate the microbiome variation in response to the dietary transition between AH and CS. Based on this knowledge, we hypothesized that the abundance of fibrolytic bacteria will increase under low-quality forage intervention and have a profound influence over the microbiota even after intervention withdrawal for a period. Furthermore, the diet-induced microbiome persistence may further influence the overall fibrolytic activity in the rumen. We seek to highlight future methods for improvement of fiber digestibility in ruminants through dietary modification.

## Materials and Methods

All animal procedures were approved by the Animal Care and Use Committee of Zhejiang University (Hangzhou, China) and were in accordance with the university’s guidelines for animal research.

### Animal Experiment and Sampling

**FIGURE 1:**
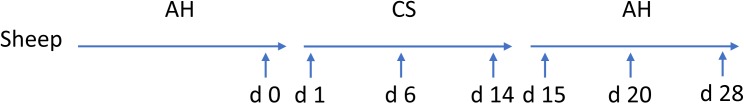
Experimental design and sample collection time points. Five sheep were tracked across dietary transition. Diet transitioned from alfalfa hay (AH) to corn stover (CS) and back to AH, with each dietary treatment lasting for 14 days. Sample collection implemented on day 0, the previous day before first transition, and days 1, 6, 14, 15, 20, and 28 subsequently.

Ten ruminally cannulated Hu Sheep (body weight = 15.1 ± 0.5 kg) were used in a crossover repeated measure design. A detailed description of this experimental design and treatments has been described previously (Xie et al., 2018). Briefly, diets were formulated according to their primary forage sources: AH or CS with forage to concentrate ratio of 6:4 (Supplementary Table S1). Animals housed individually were equally separately assigned to two treatment sequences: (1) dietary transition from AH (first period) to CS (second period) then to AH (third period); (2) dietary transition from CS (first period) to AH (second period) then to CS (third period). Each diet treatment lasted for 2 weeks before transit to the next. Animals were fed twice a day (800 h, 1600 h) with the same amount of feed according to minimum expected intake to minimize the fluctuation of feed intake. Animals had free access to water. Ruminal contents were collected before the morning feeding on the days before the first dietary transition and 1, 2, 4, 6, 9, and 14 days after the first and second dietary transition, respectively. The collected samples were then placed on ice before transporting to laboratory and stored at −20°C until further analysis. Samples from sequence AH-CS-AH were later chose to microbial analysis using next-gen sequencing, since AH-CS-AH fitted hypothesis of low quality forage intervention. Samples from sequence AH-CS-AH collected on the day before the first transition and 1, 6, and 14 days after the first and second dietary transition were subjected to DNA extraction, since the 7 samples represented early, middle, and late sections during each transition period. Setting the previous day before first transition from AH to CS as the initial point, the 7 samples were defined as days 0, 1, 6, 14, 15, 20, and 28 according to their chronological order for easier description in the results and discussion sections (**Figure 1**).

### Measurement of Microbial Enzyme Activity

The activities of CMCase and xylanase of each sample was determined according to the dinitrosalicylic acid method described by Bailey et al. (1992). CMCase sodium (Sigma-Aldrich, St. Louis, MO, United States) and xylan (Sigma-Aldrich) were used as substrates. In brief, 5 ml of homogenized rumen digesta was sonicated (20 kHz, 195 W, 5 min) using a JY92-IIN Ultrasonic Cell Mixer (Ningbo Scientz, Ningbo, China) and supernatant of each sample was collected after 14,000 × *g* centrifugation at 4°C for 10 min. Then, 0.2 ml diluted supernatant was incubated with same volume of corresponding substrates (0.01 g/ml in phosphate buffer, pH 6.0) at 39°C for 30 min. Eight-hundred microliters of DNS reagent was added to reaction mixture and the test tube was placed in boiling water bath for 5 min. The absorbance was then measured at 540 nm on a microplate reader (SpectraMax M5, Molecular Devices, San Jose, CA, United States). The enzyme activities were expressed as μmol of decomposed monosaccharides released per minute and per milliliter of each sample.

### Rumen Content Total DNA Extraction

The DNA was isolated from the selected rumen digesta samples using a cetyltrimethyl ammonium bromide based method (Sharma et al., 2003), with minor modification. In brief, precipitate centrifuged from approximately 0.8 ml of rumen digesta sample was homogenized with 1 ml cetyltrimethyl ammonium bromide lysis buffer and a 0.5 g mixture of 0.5 and 0.1 mm zirconium beads using bead-beating (FastPrep-24, M. P. Biomedicals, Santa Ana, CA, United States). The homogenized samples were incubated at 80°C for 15 min with vortexing once every 5 min. The DNA was separated with phenol-chloroform-isopentanol (25:24:1), precipitated with 1 ml isopropanol, washed several times using 75% ethanol, and then dried and dissolved in ddH_2_O. The extracted DNA was quantified using Qubit dsDNA HS Assay Kit (Invitrogen, Eugene, OR, United States) on Qubit 2.0 Fluorimeter (Invitrogen, Carlsbad, CA, United States).

### 16S rRNA Gene Sequencing and Bioinformatic Analysis

The hypervariable V3–V4 region was amplified using forward primer 5′-ACTCCTACGGGRSGCAGCAG-3′ and reverse primer 5′-GGACTACVVGGGTATCTAATC-3′ (Wang and Qian, 2009). After library construction, the pooled samples were sequenced on the Illumina HiSeq platform for paired-end reads of 250 bp. The paired-end Illumina fastq sequences were assembled using PANDAseq assembler (Masella et al., 2012) according to the overlapping region of the paired-end reads. Demultiplexed, quality control and data processing were performed using the QIIME pipeline (Caporaso et al., 2010b). Chimeric sequence detection and *de novo* OTU pick up with 0.97 identities were implemented using USEARCH and UCLUST algorithms, respectively (Edgar, 2010). The OTUs that clustered only one or two reads were removed from datasets. Taxonomy assignment of representative sequences from each OTU were performed by Ribosomal Database Project classifier (Wang et al., 2007) against its reference database (Cole et al., 2014) with confidence cutoff 0.8. Representative sequences were aligned against reference sequences using PyNAST (Caporaso et al., 2010a). The phylogenetic tree was built with the FastTree algorithm (Price et al., 2010).

### Analysis of Microbial Variation, Network and Function Prediction

The genera that significantly varied during the transition were tested by DESeq2 (Love et al., 2014). The resulting relative abundance matrix of significantly different genera were normalized by *z*-cores to assist heatmap-visualization of variation pattern within different days. The clustering was performed to relate variation pattern of each genus based on Euclidean distance. The heatmap of normalized abundance matrix was visualized using R ‘ComplexHeatmap’ package (Gu et al., 2016).

To infer the ecological interaction networks from microbiota, Molecular Ecological Network Analyses Pipeline (MENAP) adapts from Random Matrix Theory was used (Deng et al., 2012). Symmetric similarity matrix was formed by calculating Spearman’s Rho. The threshold for defining the network is set at significance of χ^2^ > 0.05, calculated based on the transition from Gaussian orthogonal ensemble to Poisson distribution of the nearest-neighbor eigenvalues. The generated network was visualized with Cytoscape (Shannon et al., 2003).

Finally, the Tax4Fun was used to predict functional genes of microorganisms (Asshauer et al., 2015). We went through all processes with default settings with closed OTU picking up against the SILVA 123 database (Quast et al., 2013), and the normalized OTU tables were used for computing metabolic capabilities using KEGG pathway reference profiles according to the MoP approach in the software. The PCA plotting and statistical hypothesis tests for pairs of samples were then performed using STAMP software (Parks et al., 2014).

### Statistical Analysis

Within-sample diversity (α-diversity) was analyzed by phyloseq (McMurdie and Holmes, 2013). Microbial diversity was determined according to the Shannon Index, dominance was presented as the Simpson index and richness of samples were calculated based on the Chao1 index and observed species. The statistical significances of difference between microbial communities at different time points were tested by Kruskal–Wallis *H*-test adjusted with false discovery rate, using R (R Core Team, 2016) facilitated with agricolae package (Mendiburu, 2016). The statistical significance was declared at *P*-value < 0.05, and trends are declared at *P*-value < 0.10.

Between-sample diversity (β-diversity) was analyzed to cluster the microbial communities with higher similarity. Dissimilarity between samples was calculated by Bray–Curtis and the resulting matrix was later ordinated according to non-metric dimensional scale analysis using phyloseq (McMurdie and Holmes, 2013), embellished with ggplot2 (Wickham, 2009). The stress of ordination of two dimensions was 0.18. The average distances between animals within each time point were calculated based on the dissimilarity matrix. The differences among samples at different time points were tested using adonis2 with vegan package (Oksanen et al., 2016) in R. Pairwise comparisons were made using permutation ANOVAs adjusted by false discovery rate on the distance matrix. Within-group dispersions were calculated and tested using ‘betadispers’ in vegan package (Oksanen et al., 2016).

The microbial communities were related with previous measured rumen fermentation parameters described previously Xie et al. (2018) with redundancy analysis using vegan package (Oksanen et al., 2016). The top 15 significant different genera were used as community matrix, and rumen fermentation variables were introduced as environmental constraint variables. Monte Carlo Permutation was applied to test the influence of environmental variables on the microbial composition. The significance was considered for *P*-values < 0.05. Multivariate analysis by linear models (MaAsLin) (Morgan et al., 2012) was also performed to show the potential association between fermentation metadata with microbial community, allowing detection of the effect of one metadata deconfounding the effects of others.

The LDA of effect size algorithm (Segata et al., 2011) was performed for estimating the effect size of species that attribute to the difference of samples in each day. The threshold of LDA score was set at default value 2.0, so that the abundance of species with at least 100-fold change were considered significant.

## Results

### Enzyme Activities Variation

Both CMCase and xylanase catalytic activities increased (*P* < 0.05) when forage transited from AH to CS (**Table 1**). After forage transited back to AH, the enzyme activities decreased (*P* < 0.05), but remained significantly higher than day 0 (*P* < 0.05).

**Table 1:** Temporal variation of enzyme activities in the sheep rumen.

Time	Forage	Enzyme activity (U/mL)	
		CMCase^1^	Xylanase
Day 0	AH	0.245^a^	0.402^a^
Day 1	CS	0.309^bc^	0.680^bc^
Day 6		0.354^c^	0.825^c^
Day 14		0.378^c^	0.868^c^
Day 15	AH	0.318^bc^	0.708^bc^
Day 20		0.271^ab^	0.506^b^
Day 28		0.296^b^	0.539^b^
SEM		0.021	0.054
*P*-value		<0.01	<0.01

*^1^CMCase, Carboxymethyl cellulase. ^a–c^Means with different superscript letter differ (P < 0.05) among the sampling days.*

### Diversity of Ruminal Microbial Communities Through Dietary Transitions

After concatenation and quality control, a total of 1,412,315 clean reads (35,308 ± 412 per sample) were obtained. The *de novo* OTU clustering resulted in an average of 1,156 OTUs with a Good’s coverage of 98.7% across all samples. Alpha diversity was significantly different (*P* < 0.05) across all time points, according to Chao1 and Shannon indexes of diversity and richness in microbial communities, but not for Simpson (**Table 2**). For the number of observed species, Chao1 and Shannon indexes increased numerically after the first dietary transition from AH (day 0) to CS (days 1–14) and reached their peak on day 15, the first day after second transition from CS to AH (days 15–28). At the end of post-AH treatment (day 28), the diversity was significantly higher than that at day 0 (*P* < 0.05).

**Table 2:** Statistical summary for alpha diversity of ruminal microbial community.

Sample	chao1	Observed species	Shannon	Simpson	Goods coverage (%)
Day 0	1255^c^	1040^c^	5.35^b^	0.986	98.63^a^
Day 1	1252^c^	1047^c^	5.40^b^	0.986	98.63^a^
Day 6	1416^b^	1177^ab^	5.56^ab^	0.988	98.49^ab^
Day 14	1480^ab^	1233^a^	5.58^ab^	0.987	98.35^b^
Day 15	1498^a^	1250^a^	5.62^a^	0.988	98.48^b^
Day 20	1404^b^	1154^b^	5.51^ab^	0.987	98.34^ab^
Day 28	1442^ab^	1192^ab^	5.61^a^	0.990	98.50^ab^
SEM	29	20	0.09	0.002	0.00
*P*-value	<0.01	<0.01	0.04	0.11	<0.01

*^a–c^Means with different superscript letter differ (P < 0.05) among the sampling days.*

### Taxonomic Composition of the Rumen Microbiota in the Hu Sheep

Overall, 17 phyla were detected, with 12 of them identified in the rumen of all sheep across all time points (Supplementary Figure S1). *Bacteroidetes* and *Firmicutes* were dominant across all rumen bacterial communities, accounting for approximately 90% of total sequences. The phyla *Proteobacteria*, *Actinobacteria*, *Spirochaetes*, *Fibrobacteres*, *Tenericutes*, *Candidatus Saccharibacteria*, and *Synergistetes* were less abundant, accounting for 0.1–10% of the total sequences. A total of 102 genera were detected at the genus level, with the top 20 most abundant genera shown in Supplementary Figure S2. The rank of each genus varied across different points in time. On day 0, the most abundant genus was *Prevotella*, accounting for 23.3% (± 1.81, standard error) of total bacteria, followed by *Butyrivibrio* (2.15 ± 0.57%), *Succiniclasticum* (1.52 ± 0.26%), *Ruminococcus* (1.28 ± 0.25%), and *Olsenella* (0.83 ± 0.20%). On day 14, *Saccharofermentans* (2.40 ± 0.23%) increased to the second most abundant genera after *Prevotella* (15.62 ± 0.78%), followed by *Succiniclasticum* (1.82 ± 0.16%)*, Ruminococcus* (1.32 ± 0.12%), and *Butyrivibrio* (1.16 ± 0.13%). On day 28, the *Prevotella* (25.60 ± 1.40%) was still dominant over all the genera, followed by *Succiniclasticum* (2.78 ± 0.18%), *Butyrivibrio* (1.67 ± 0.21%), *Ruminococcus* (1.02 ± 0.16%), and *Paraprevotella* (0.88 ± 0.30%).

### Compositional Variation of Ruminal Microbial Communities During Dietary Transition

Among 102 detected genera, the relative abundances of 47 genera were significantly altered during the dietary transition. These 47 genera were grouped into four main clusters (Supplementary Figure S3). The first two microbial clusters changed significantly according to the forage type. In these two categories, *Prevotella*, *Campylobacter*, *Succinivibrio*, *Eubacterium*, *Erysipelotrichaceae incertae sedis*, and *Coprococcus* were more abundant when animals consumed AH, while *Streptococcus*, *Clostridium XIVb*, *SR1_genera_incertae_sedis*, *Rhizobium*, *Ethanoligenes*, *Saccharofermentans*, *Rhodococcus*, *Methylobacterium*, *Sphaerochaeta*, *Mycobacterium*, *Kalstia*, and *Saccharibacteria genera incertae sedis* were more abundant when animals were fed on CS. The relative abundance of the third category genera such as *Succiniclasticum*, *Pyramidobacter*, *Candidatus Endomicrobium*, and *Fibrobacter* increased when diet was transitioned from AH to CS, while their relative abundance was maintained or even increased when diet was transitioned from CS back to AH. However, the relative abundance of *Olsenella*, *Clavibacter*, *Atopobium*, *Syntrophococcus*, and *Desulfobulbus* decreased after forage was transited from AH to CS but was lower than the pre-CS treatment even after forage was transitioned back to AH.

### Microbial Community Shift Affected by Dietary Transition and Individuality

**FIGURE 2:**
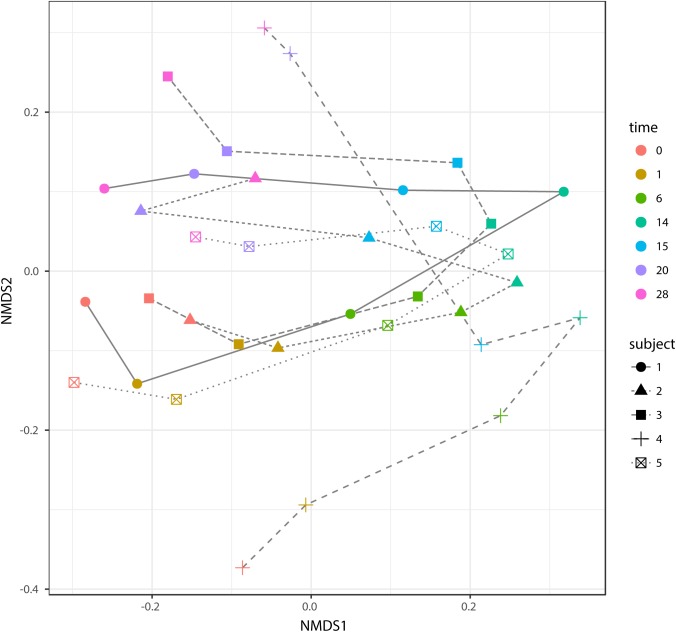
Non-metric dimensional analysis (NMDS) of beta-diversity and Bray–Curtis similarity and trajectory of each microbial community. Samples belonged to different subjects in different time points and were indicated with different shapes and colors. The trajectory of each microbial community was represented using different line types. Scatters on days 0 and 28 were separately clustered (*P* < 0.05, adonis2).

**FIGURE 3:**
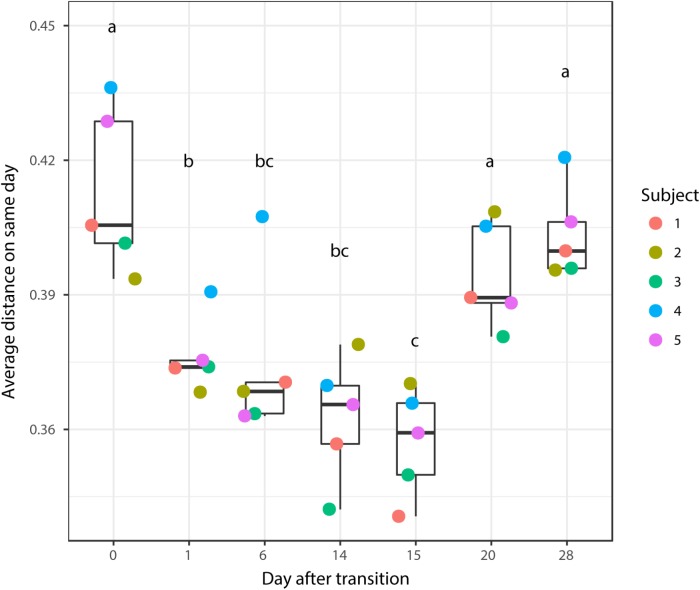
Average distance between the rumen microbial community from the present day and the previous day. Changes in the average distance of the rumen microbial community between the five animals were plotted in the 1-dimensional scale against transition time points. Each colored point represented average distance from one animal to other animals. Different letters denote the significant difference of means (*P* < 0.05) among different times.

According to non-metric multidimensional scaling analysis visualized in **Figure 2**, the microbial communities were closely clustered according to sampling dates rather than by individuals. In addition, the samples on the first day of transition (days 1 and 15) showed higher similarity to the day before transitions (days 0 and 14, *P* = 0.670 and 0.343, respectively), while the samples from the 6th day after transition (days 6 and 20) were much closer to the samples from 14th day after transition (days 14 and 28, *P* = 0.081 and *P* = 0.485, respectively, **Table 3**). After the second dietary transition back to AH, the microbial community on days 0 and 28 remain separately clustered. The adonis2 variation analysis of Bray–Curtis dissimilarity confirmed that the two microbial communities were compositionally different (*P* < 0.01, **Table 3**). Homogeneity of group-dispersion analysis indicated that diets had a significant influence over within-group dispersion (*P* < 0.05). **Figure 3** demonstrated the intra-animal variation based on 1-day average Bray–Curtis dissimilarity between the animals. The average distance between animals decreased from days 0 to 14 (*P* < 0.05) after their diet transitioned from AH to CS and then increased (*P* < 0.05) after diet transitioned back to AH. Furthermore, in the ranking of average distance between each animal, a representative of individuality effects over the microbial community was not different between days 0 and 28 (**Figure 3**).

**Table 3:** Pairwise comparisons on Bray–Curtis distance of each microbial community.

*P*-value	Day 0	Day 1	Day 6	Day 14	Day 15	Day 20
Day 1	0.670	–	–	–	–	–
Day 6	0.015^∗^	0.015^∗^	–	–	–	–
Day 14	0.015^∗^	0.015^∗^	0.081	–	–	–
Day 15	0.015^∗^	0.015^∗^	0.016^∗^	0.343	–	–
Day 20	0.015^∗^	0.015^∗^	0.015^∗^	0.015^∗^	0.015^∗^	–
Day 28	0.028^∗^	0.015^∗^	0.015^∗^	0.016^∗^	0.015^∗^	0.485

*^∗^Significantly different between the sampling days (P < 0.05, adnois2) according to Bray–Curtis dissimilarity.*

### Bacteria Increased via CS Intervention and Persisted After Intervention Withdraw

**FIGURE 4:**
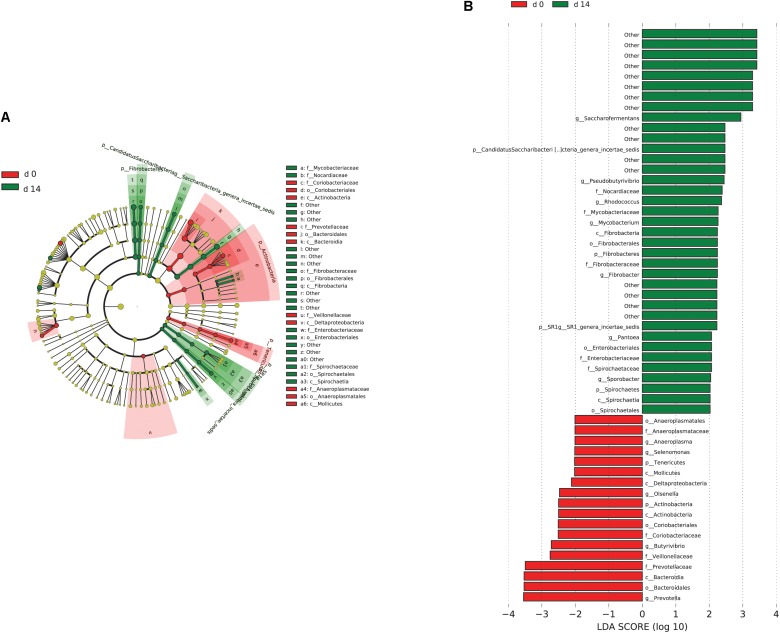
Microbial community differences between AH (day 0) and CS (day 14). **(A)** LEfSe cladogram of the microbial communities compared between AH (day 0) and CS (day 14). Differences are represented in the color of the group, where taxa are most abundant. Red: taxa abundant in AH (day 0); Green: taxa abundant in CS (day 14). **(B)** Histogram of linear discriminant analysis (LDA) scores computed for each taxon ranging from phylum to genus. The LDA scores represented the difference in relative abundance with exponent fold change of 10 between two communities. The taxa statistically different explain the difference between. Group ‘other’ represented genera that were unable to be classified.

**FIGURE 5:**
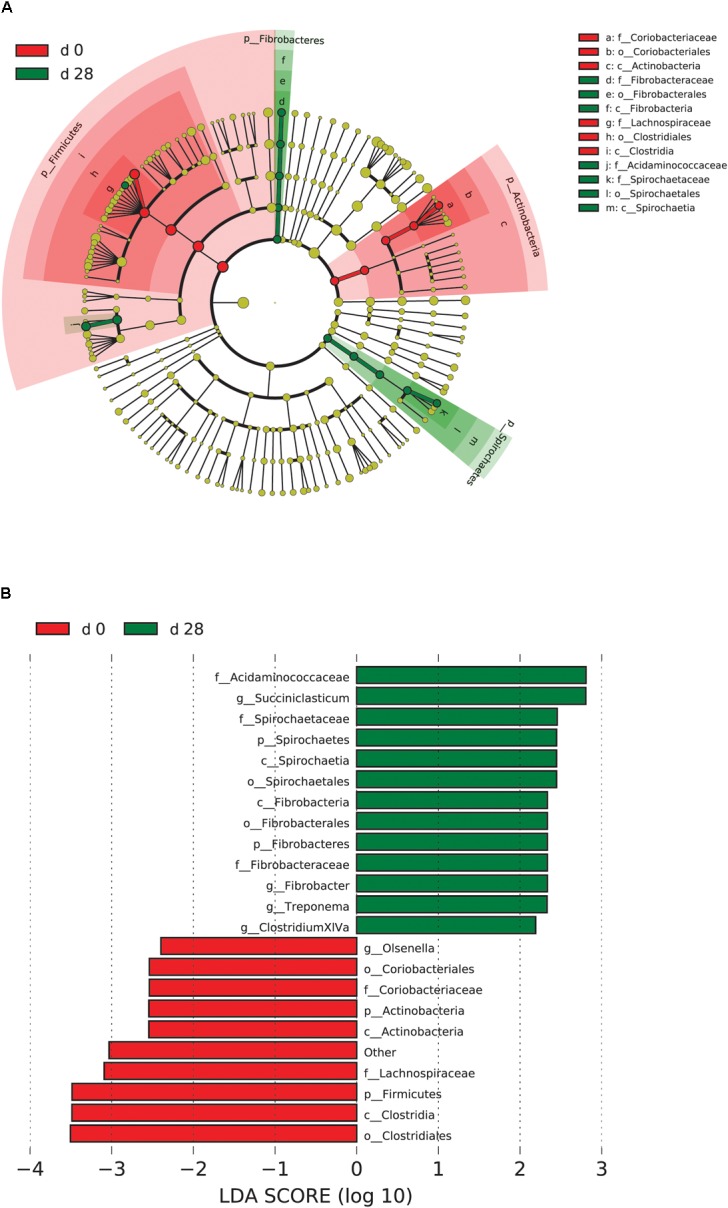
Microbial community differences between AH (day 0) and post intervention AH (day 28). **(A)** LEfSe cladogram of the microbial communities compared between AH (day 0) and post intervention AH (day 28). Differences are represented in the color of the group, where taxa are most abundant. Red: taxa abundant in AH (day 0); Green: taxa abundant in post AH (day 28). **(B)** Histogram of LDA scores computed for each taxon ranging from phylum to genus. The LDA scores represented the difference in relative abundance with exponent fold change of 10 between two communities. The taxa statistically different explain the difference between. Group ‘other’ represented genera that were unable to be classified.

**FIGURE 6:**
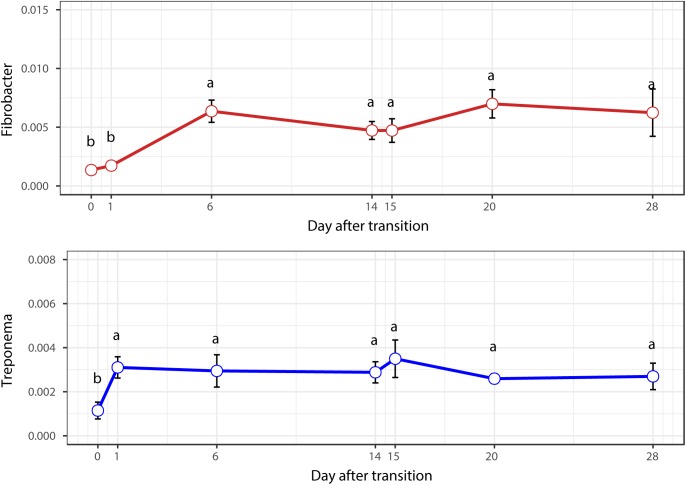
Temporal variation of the relative abundances of *Fibrobacter* and *Treponema.*

When the microbial communities were compared between AH (day 0) and CS (day 14), the most differentially abundant bacterial taxa in animals fed with CS diet on day 14 belong to the genera *Saccharofermentans*, *Pseudobutyrivibrio*, *Rhodococcus*, *Mycobacterium*, *Fibrobacter*, *Pantoea*, and *Sporobacter*, while genera *Prevotella*, *Butyrivibrio*, *Olsenella*, *Selenomonas*, and *Anaeroplasma* were more abundant on day 0 under AH diet (**Figure 4**). The genera *Saccharofermentans* and *Prevotella* were the taxa that weighted most to the differences between communities, with an absolute LDA score factors approximately 3. When the microbial communities were compared between AH (day 0) and AH post intervention (day 28), *Succiniclasticum*, *Fibrobacter, Treponema*, and *Clostridium XIVa* were the genera statistically significantly abundant under post intervention AH diet (day 28), while *Olsenella* was mostly relatively abundant in AH diet (day 0) (**Figure 5**). The relative abundance of *Fibrobacter* and *Treponema* were highly correlated with acetate to propionate ratio and acetate molar proportion respectively (Supplementary Figure S4). Of all the genera significantly variated across dietary transition, *Fibrobacter* and *Treponema* were the only taxa that were promoted under the CS feeding regime but persisted even 14 days after forage transition back to AH (**Figure 6**). In contrast, the relative abundance of *Olsenella* was reduced under CS but not recovered back to AH.

### Inference Co-occurrence Network Centered by *Fibrobacter*

**FIGURE 7:**
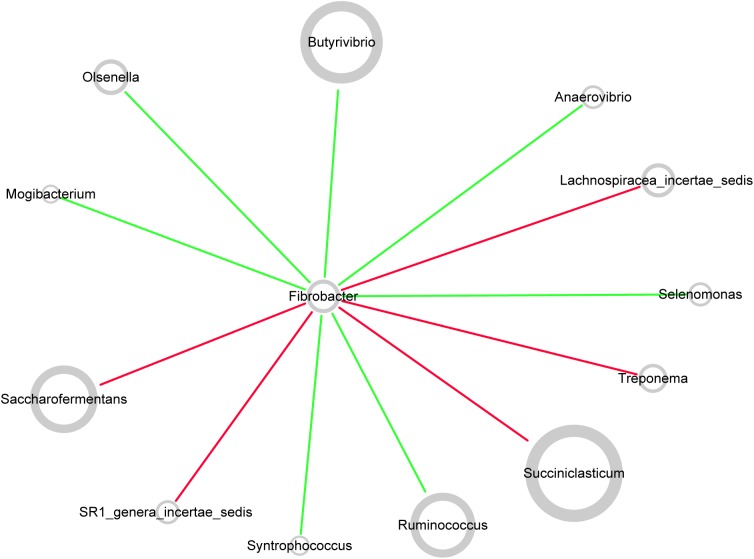
Co-occurrence network of rumen microbiota centered by *Fibrobacter.* Each node in the network represented a genus from the microbiome, and each colored edge represents a pairwise co-occurrence or interaction. The relative abundances of each genera were represented by nodes sizes. The edges that positively linked two nodes are highlighted in red, and edges that negatively linked two nodes are highlighted in green.

Network topology revealed correlations between genera in microbiota. All curves of the network were connectivity fitted with the power-law model (*R*^2^ = 0.541). The sub-network centered by *Fibrobacter* is visualized in **Figure 7**. The picture demonstrated positive correlation between *Fibrobacter* and *Succiniclasticum*, *Saccharofermentans*, *Lachnospiraceae incertae sedis*, *Treponema*, or *SR1 genera incertae sedis* and negative correlation between *Fibrobacter* and *Butyrivibrio*, *Ruminococcus*, *Olsenella*, *Anaerovibrio*, *Selenomonas*, *Syntrophococcus* or *Mogibacterium*.

### Metagenomic Function Prediction

**FIGURE 8:**
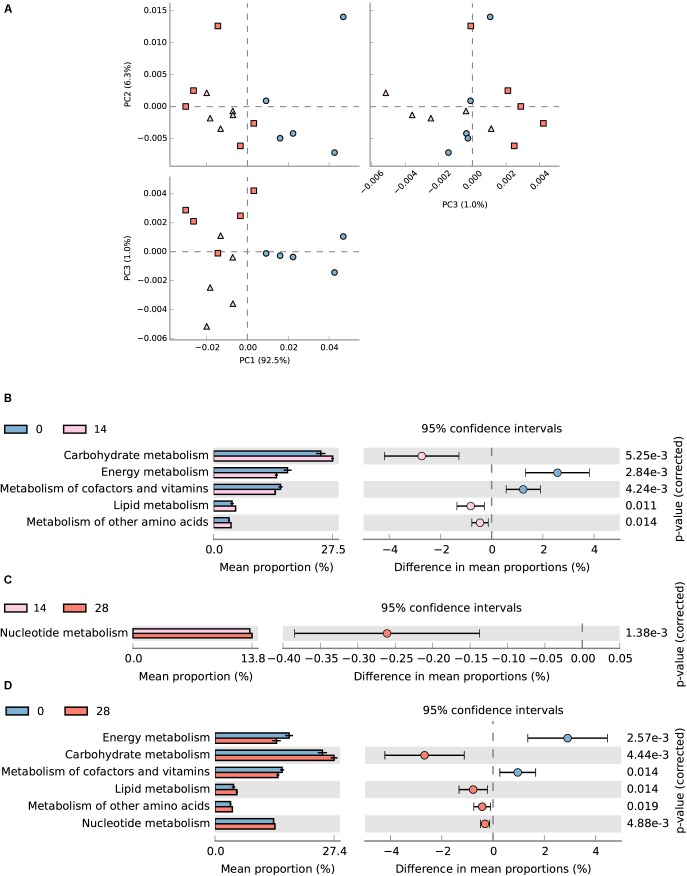
Predicted metagenomic difference between days 0 and 28. Samples of predicted metagenome from days 0 and 28 were colored using blue and red, respectively. **(A)** Principle components analysis of predicted metagenome between days 0, 14, and 28. **(B)** The metabolism pathways significantly different between samples from days 0 and 14 at KEGG level 2. **(C)** The metabolism pathways significantly different between samples from days 14 and 28 at KEGG level 2. **(D)** The metabolism pathways significantly different between samples from days 0 and 28 at KEGG level 2. Pathways were sorted in descending order based on effect factor.

In total, 54.7 (± 0.69)% of OTUs mapped to the SILVA database 123 were successfully assigned to KEEG orthologs (KO) and their corresponding pathways (at level 2) using Tax4Fun (Asshauer et al., 2015). The assigned KEGG pathways of samples from days 28 and 14 were separated from day 0 according to PCA plotting (**Figure 8A**). Diet transition from AH (day 0) to CS (day 14) increased the KO abundance in carbohydrate metabolism (from 13.41 to 14.89%), lipid metabolism (from 2.31 to 2.75%) and metabolism of other amino acids (from 1.94 to 2.19%), but decreased energy metabolism (from 9.27 to 7.88%) and metabolism of cofactors and vitamins (from 8.39 to 7.72%) (*P* < 0.05, **Figure 8B**). The statistical comparison between days 0 and 28 demonstrated that the CS intervention significantly decreased the KO abundance in energy metabolism (from 9.27 to 7.51%), and metabolism of cofactor and vitamins (from 8.39 to 7.68%), but increased carbohydrate metabolism (from 13.41 to 14.52%), lipid metabolism (from 2.31 to 2.67%), and metabolism of other amino acids (from 1.94 to 2.12%) (*P* < 0.05, **Figure 8D**). No significant difference was found between days 14 and 28 except for the increased nucleotide metabolism (from 6.57 to 6.70%) (*P* < 0.05, **Figure 8C**).

## Discussion

Diet is one of the major factors that drives the change of rumen microbiota along with various environmental factors (De Filippo et al., 2010; Walker et al., 2011; Carmody et al., 2015). Consistent with earlier reports in ruminants fed a forage-based diet (Huws et al., 2016), we identified high abundances of *Prevotella, Butyrivibrio*, *Ruminococcus*, *Succiniclasticum* and *Paraprevotella*, *Fibrobacter* and *Olsenella* in all collected samples in the current study, suggesting that these taxa play essential roles in occupying niches in rumen ecology and in the degradation of forage-based diets.

### Shifts in Rumen Microbiota in Response to Dietary Transition From AH to CS

When shifting diets from AH to CS, we observed a significant alteration in microbial composition. Of the most dominant genera detected in our study, *Prevotella* and *Butyrivibrio* were significantly decreased in line with a recent study to compare microbial colonization between AH and rice straw (Liu et al., 2016). These two genera compose a wide range of functionalities to degrade both carbohydrates and proteins (Krause et al., 2003). *Prevotella* is also combined with a very diverse range of isoforms to degrade the hemicellulose matrix formed by pectins, hemicellulose and peptides (Rubino et al., 2017). The increased relative abundance of both species may be partially associated with high compositions of neutral detergent-soluble and crude protein in AH.

Out of the eight significantly increased genera with LDA effect size factors larger than two, *Fibrobacter* is commonly identified as one of the most abundant and efficient bacterial degraders of lignocellulosic material in the bovine rumen (Ransom-Jones et al., 2012) and is highly abundant in the rumen of animals fed with low-quality forages, is highly efficient in degrading crystalline cellulose, and shows a high ability to solubilize plant cell wall polysaccharides (Béra-Maillet et al., 2009). Other bacteria including *Saccharofermentans* (Nyonyo et al., 2014), *Pseudobutyrivibrio* (Grilli et al., 2013), *Sporobacter* (Grech-Mora et al., 1996), and *Treponema* (Bekele et al., 2011) that increased in CS are also known to be involved in fiber degradation. These results were basically consistent with previous reports of forage effects over rumen microbial communities in dairy cows (Zhang et al., 2014). Although *Rhodococcus*, *Mycobacterium*, and *Pantoea* have been identified in many studies, their functions in the rumen were rarely described. Some members belonging to these genera have been reported to have lignin decomposition functions (Janusz et al., 2017), indicating their potential role in stover degradation in the rumen. Therefore, feeding of animals with CS facilitated the growth of lignocellulose degraders in the rumen and shifted the overall microbiota to a more fibrolytic degradation-efficient community compared to AH feeding.

### Microbial Persistence During Dietary Transition From CS Back to AH

When the diet was transitioned from CS back to AH, the communities were pushed in another direction rather than back to their original composition (**Figure 2**). Comparison of the original AH (day 0) and post-intervention AH (day 28) communities indicated that *Fibrobacter* and *Treponema* were the only genera that increased when forage was transitioned to CS but showed persistence to the transition back to AH. The apparent digestibility of NDF and ADF, total VFA concentration and acetate molar proportion were slightly increased, though the difference was not statistically significant (Xie et al., 2018). However, the absolute concentration of acetate at day 28 were significantly higher than that at day 0 (*P* < 0.05). Acetate is a major end-product of cellulose fermentation. The increased concentration is indicative of increased fiber degradation in the rumen. Combined with the increased rumen CMCase and xylanase activities and predicted result of increased microbial functions involved in carbohydrate metabolism, we speculate that the intervention of the CS diet could facilitate the fibrolytic bacteria and enhance the ruminal fibrolytic potential for a period, even after the intervention withdrawal. In this case, the system failed to return to the original AH state in 2 weeks, possibly because *Fibrobacter* and *Treponema* are well suited for both forage-based dietary feeds, and they cannot easily be outcompeted when the system returns to its original AH conformation. Meanwhile, other special groups such as the genus *Olsenella* were eroded beyond their ability to recover. In a study using NMR to monitor wheat straw degradation by *Fibrobacter*, Forano et al. (2008) found that *Fibrobacter* was able to use reversible carbohydrate metabolism pathways to synthesize oligosaccharides that were stored as intracellular carbohydrate reserves, and facilitated its ability to rapidly adapt to sudden environmental changes. For *Treponema*, its persistence was likely benefiting from the cross-feeding network centered by *Fibrobacter* (**Figure 7**). Numerous studies have shown that *Fibrobacter* and *Treponema* shared mutual interaction (Debroas and Blanchart, 1993; Fondevila and Dehority, 1996; Shinkai et al., 2010), in which *Fibrobacter* relies on *Treponema* to break down the interlaced hemicellulose to easily access to fiber components, and in return, it provides cellulose hydrolysates to *Treponema*. In addition, *Treponema’*s ability to utilize pectin that abundant in AH may further support its persistence in post-intervention AH period (Liu et al., 2015).

### Variation in Microbial Diversity During Dietary Transition

Notably, in addition to the genera persisted after CS intervention, the diversity of rumen microbiota was reduced during CS treatment and did not increase upon return to AH. The diversity, dominance and richness are regarded as the key elements that can have a major effect on the functionality of the rumen (Weimer, 2015). Along with the increasing diversity and richness of the microbial community after transitioning to a CS-based diet, the inter-animal differences decreased. The narrowing individual difference and increasing diversity from AH to CS suggested the rumen microbiome became more phylogenetically diverse but convergent, driven by CS. This may be partially related to the suppression of species domination under low nutrient conditions and drive the microbial community into a more similar and specific functional repository to break down more recalcitrant resource compounds (Marino et al., 2014). The decrease of inter-animal differences may also be an indication of decreases in microbial redundancy, since highly divergent compositions of microbiota across individuals fulfill the same functions and ensure individuality (Lozupone et al., 2012). Since catabolic redundancy is skewed heavily toward the most abundant and common substrate, such as soluble carbohydrate highly abundant in AH (Weimer, 2015), it is likely that the reduction of the abundance of general microbes competing for common substrates and the increase of less competitive functional specialized microbes involved in typically less abundant substrate reduce the individual differences. Although there is no clear evidence, the persistence of microbial α-diversity along with the identified fibrolytic bacteria may not be merely coincidence. The increased richness and diversity of microbiota may possibly relate to persistence of fibrolytic bacteria.

### Individualized Adaptation in Terms of Overall Microbial Profiles

Another notable point is the effect of individuality on microbial community change during forage transition. Although the closely clustered samples were influenced by diet rather than by individual, the rank of similarity distances between each animal remained the same between days 0 and 28 (**Figure 3**). Furthermore, the magnitude of reaction to and after CS intervention, and the speed and extent of recovery to the pretreatment state, suggested that the resilience and adaptation of microbiota varies across individuals. Obviously, the diet dominates the effects of host genotype in shaping the microbial structure according to our observation (Carmody et al., 2015), and the inter-animal variability linked to the impact of host genotype cannot ever get beyond (Jami et al., 2013; Henderson et al., 2016). To achieve the optimal outcome using dietary intervention, individual variation should also be considered.

Finally, to our knowledge, this is the first time, rumen microbial hysteresis has been described, though this characteristic of microorganisms has been previously reported in mice models (Carmody et al., 2015; Thaiss et al., 2016). Long-term or lifetime low-quality forage treatment for animals is not a wise choice for productivity because of the inherently low nutrients in low-quality forage cannot support the faster growth and better animal performance. Feeding animals with high-quality forage while using low-quality forage intervention as a booster can avoid nutrition deficits. Our results indicated that microbiome is rapidly altered by diet and 2 weeks is enough time to shift the rumen microbial composition. This was evident in **Figure 2** and **Table 3** that the microbial dissimilarity distance was not significant between the “middle” and “end” points of the diet period. However, it may be difficult to conclude that the microbiome has reached a new equilibrium, since major variation of bacteria has shifted back by diet while residual variation remains due to the transitions. Additional time may be needed to observe longer transition. It is also noticeable that although, observation of bacteria persistence can be performed by comparing between samples collected pre- and post-intervention, which also leave out difference among individuals. In this design, without comparison to animals feeding AH diet across all experiment, we can’t take into account any longitudinal variation over time during transition. Besides, additional work is needed to test the underlying source of hysteresis and investigate whether it is due to dynamic adaption strategy for microbiota to remember their past dietary exposures or whether it is mainly through reconstruction of bacterial interaction networks. In addition, both short-term interventions and long-term interventions with various lengths are worth considering and comparing to investigate optimal intervention time for microbial modification, which remains a fascinating area for future inquiry.

## Conclusion

Compared to AH, feeding sheep with CS stimulates the relative abundance of fibrolytic bacteria in the rumen. Short-term intervention of low-quality forage had a significant impact on rumen microbial structure even after the intervention withdrawal. The major difference was caused by the increase of the genera *Succiniclasticum*, *Fibrobacter*, *Treponema*, and *Clostridium XIVa*, among which *Fibrobacter* and *Treponema* were the taxa that stimulated under CS but persisted after the diet transitioned back to AH for 2 weeks observation. Along with increased ruminal CMCase and xylanase activities and enrichment of carbohydrate metabolism pathways, these results suggested a degree of acclimatization and persistence of fibrolytic bacteria and a potential increase of fiber digestibility in the rumen. Based on the hysteresis characteristic of *Fibrobacter* and *Treponema*, repeated interventions using low-quality forage while feeding animals with high-quality forage may be a promising strategy to enhance the hosts’ fibrolytic digestibility and for promoting the livestock industry in the long run.

## Author Contributions

XX, JL, and JW conceived and designed the study. XX performed both animal feeding and laboratory experiments, analyzed the sequencing data, interpreted the data, prepared the figures and tables, and wrote the manuscript. CY, LG, JL, and JW helped interpret the data and write and revise the paper. All authors read and approved the final manuscript.

## Conflict of Interest Statement

The authors declare that the research was conducted in the absence of any commercial or financial relationships that could be construed as a potential conflict of interest.

## Abbreviations

AH: alfalfa hay
CMCase: carboxymethyl cellulase
CS: corn stover
LDA: linear discriminant analysis
OTU: operational taxonomic units
