# Supplementary material for: Persistence of Cellulolytic Bacteria Fibrobacter and Treponema After Short-Term Corn Stover-Based Dietary Intervention Reveals the Potential to Improve Rumen Fibrolytic Function
Source: Front Microbiol. 2018 Jun 26;9:1363. doi: 10.3389/fmicb.2018.01363 (PMC6029512; doi:10.3389/fmicb.2018.01363)
Supplement: Supplementary file 1 [file Table_1.docx]

Supplementary Material

**Persistence of cellulolytic bacteria *Fibrobacter* and *Treponema* after short-term corn stover-based dietary intervention reveals the potential to improve rumen fibrolytic function**

**X. Xie,^1^ C. L. Yang,^1^ L. L. Guan,^1,2^ J. K. Wang,^1^ M. Y. Xue,^1^ J. X. Liu^1*^**

***Correspondence:** Dr. Jianxin Liu: [liujx@zju.edu.cn](mailto:liujx@zju.edu.cn)

Table S1. Ingredients and chemical composition of experimental diets^1^

| Item | AH | CS |
| --- | --- | --- |
| Ingredients, g/100 g of dry matter |  |  |
| Alfalfa hay | 60.0 | — |
| Corn stover | — | 60.0 |
| Concentrate mixture^2^ | 40.0 | 40.0 |
| Composition, g/100 g of dry matter |  |  |
| Organic matter | 87.8 | 89.0 |
| Crude protein | 17.9 | 9.36 |
| Neutral detergent fiber | 33.3 | 54.6 |
| Acid detergent fiber | 19.6 | 37.2 |

^1^AH: alfalfa hay-based diet; CS: corn stover-based diet

^2^Contained (g/100 g, DM basis): 45 ground corn grain, 20 cottonseed meals, 15 soybean meals, 15 wheat bran, 2 salts, 1 NaHCO_3_, 2 Ca_3_(PO_4_)_2_

Figure S1 Ruminal microbial community phylum structure during dietary transition

Figure S2. Relative abundance of the top 20 most abundant genera at each time point

Figure S3 Rumen microbial community variation during dietary transition

The rows represented the samples from individual animals in different days and columns corresponded to the genera significantly variated during dietary transition. The relative abundances were normalized by z-scores for easier visualization of variation pattern of each genus across different time points and colored in the map. Genera was clustered according to the UPGMA (Unweighted Pair Group Method with Arithmetic Mean) calculated based on Spearman`s correlation of scaled abundance.

Figure S4. Triplot of the redundancy analysis of the microbial community related to rumen fermentation parameters

The genera were indicated by triangle arrows with dashed lines, and rumen fermentation parameters were indicated by triangle arrows with solid lines. Samples from different time points were presented in different colors.
