# Supplementary figures and images for: Persistence of Cellulolytic Bacteria Fibrobacter and Treponema After Short-Term Corn Stover-Based Dietary Intervention Reveals the Potential to Improve Rumen Fibrolytic Function
Source: Front Microbiol. 2018 Jun 26;9:1363. doi: 10.3389/fmicb.2018.01363 (PMC6029512; doi:10.3389/fmicb.2018.01363)

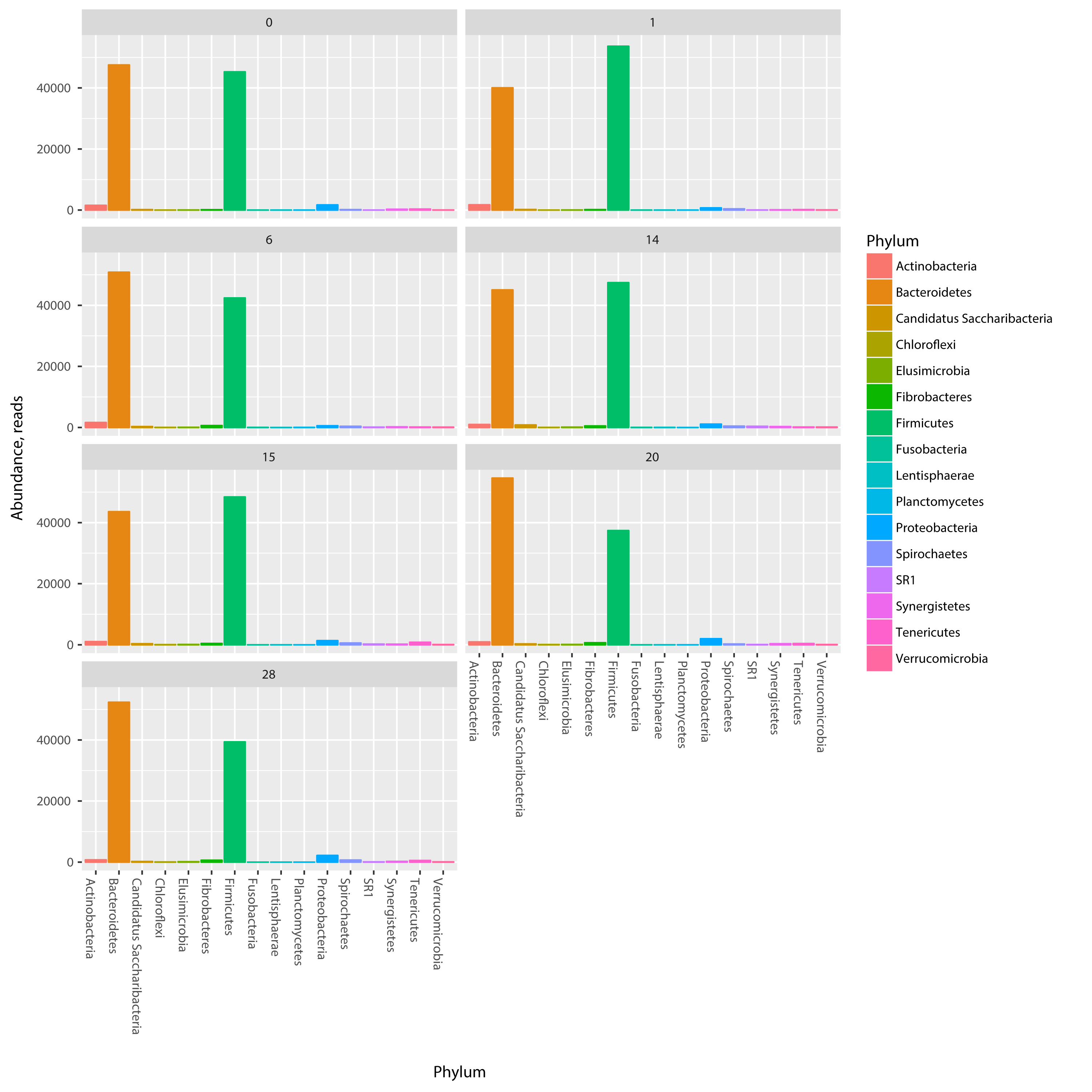

Supplement: Supplementary file 2 [file Image_1.TIF]

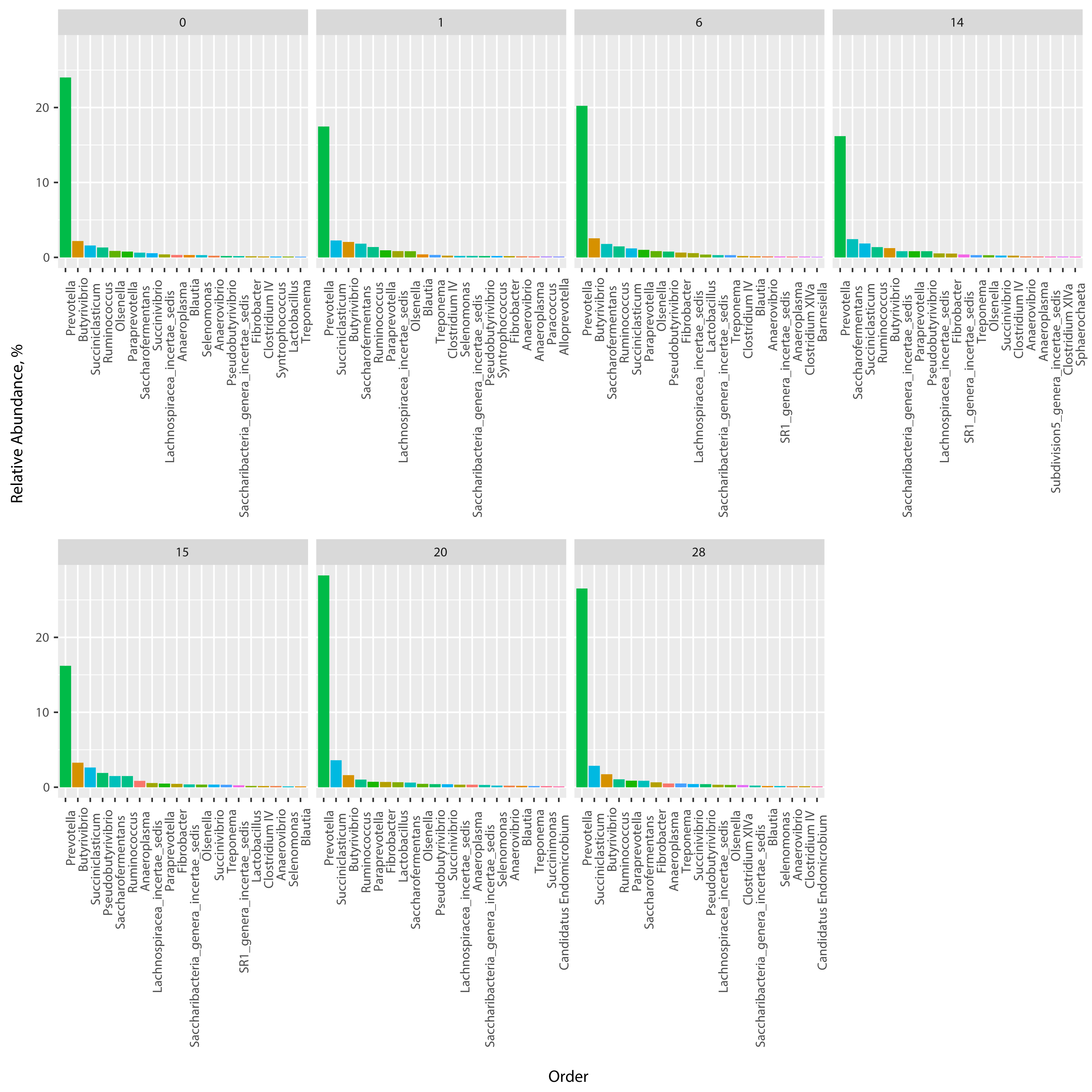

Supplement: Supplementary file 3 [file Image_2.TIF]

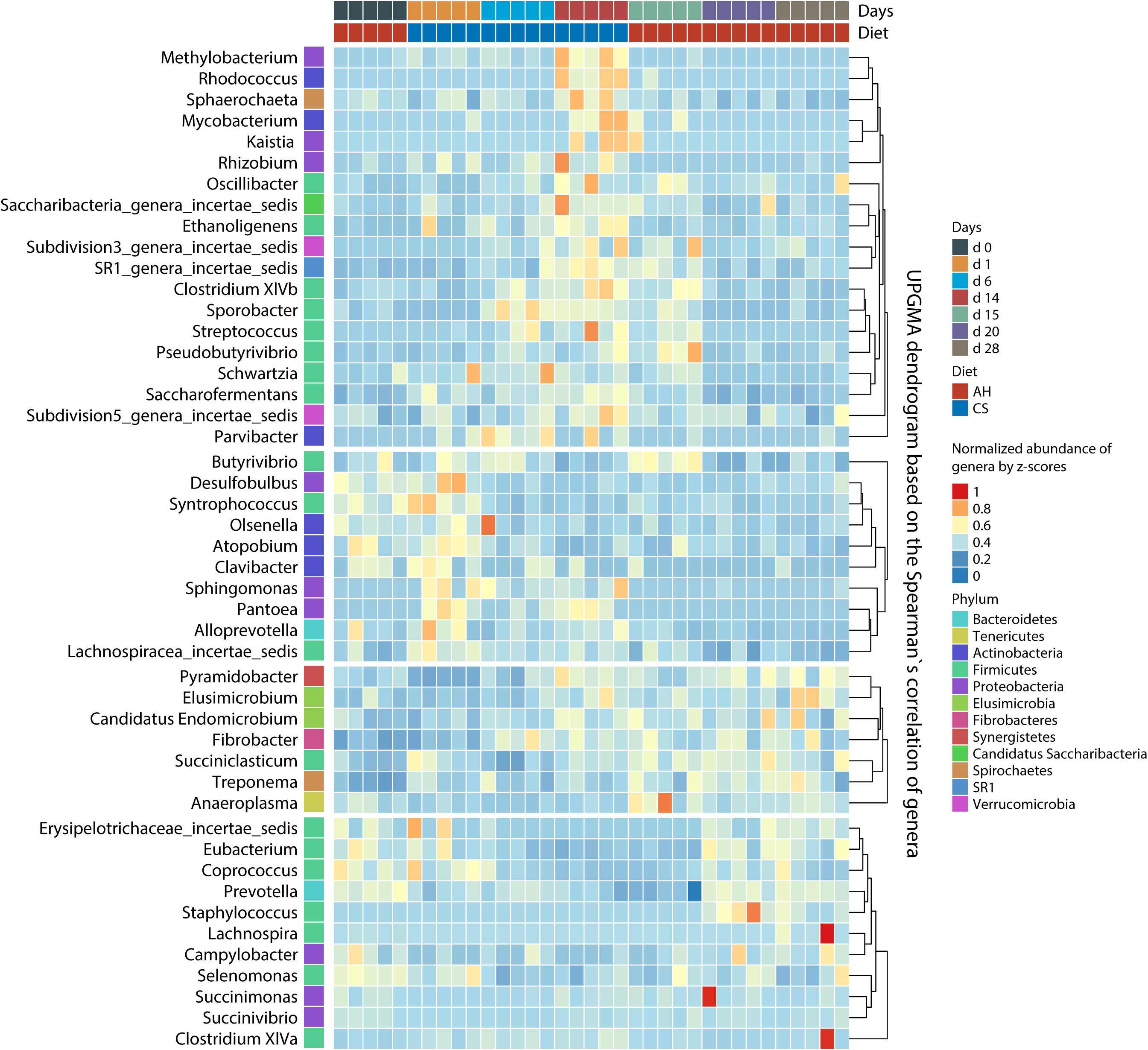

Supplement: Supplementary file 4 [file Image_3.TIF]

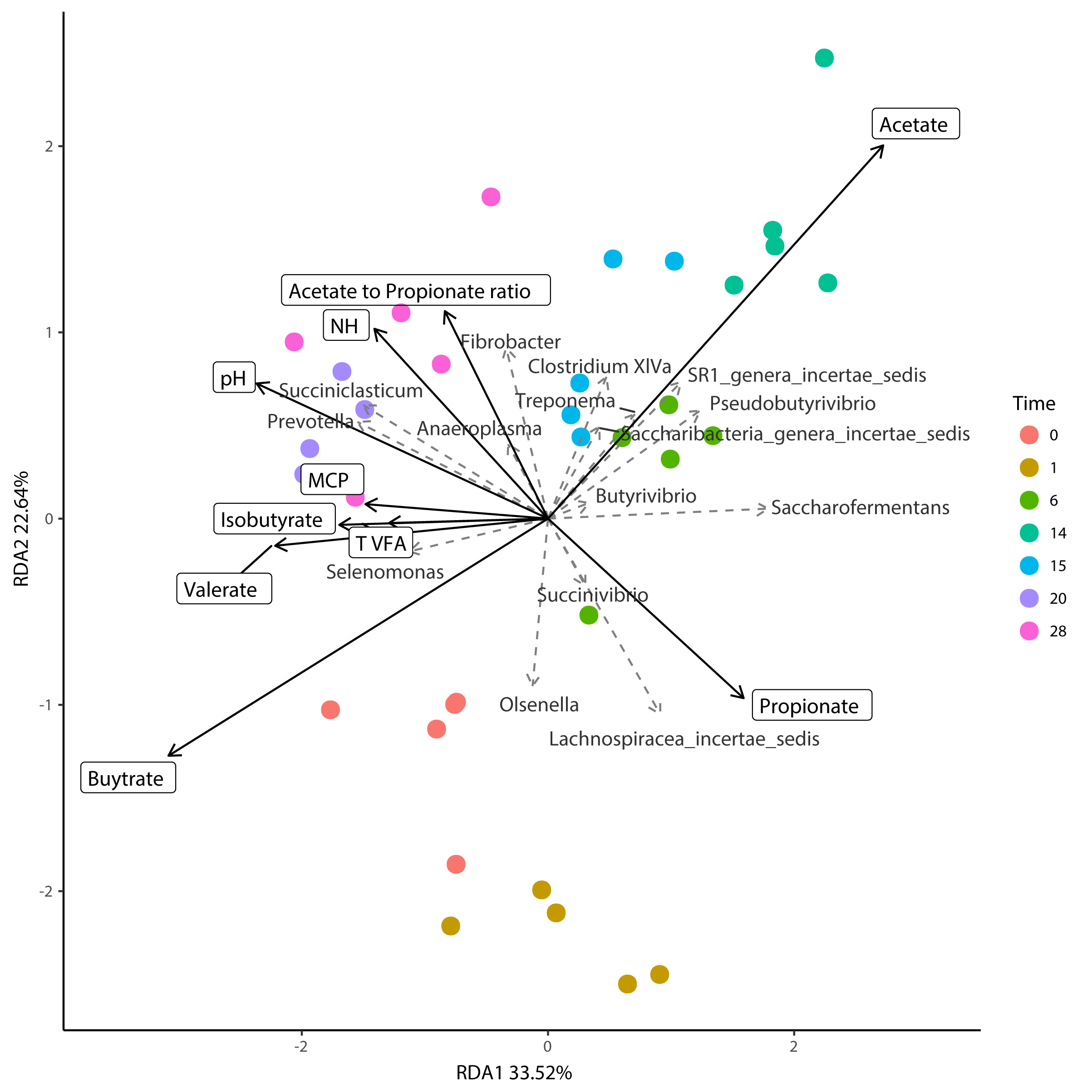

Supplement: Supplementary file 5 [file Image_4.TIF]
